# Enhanced Boron Tolerance in Plants Mediated by Bidirectional Transport Through Plasma Membrane Intrinsic Proteins

**DOI:** 10.1038/srep21640

**Published:** 2016-02-23

**Authors:** Kareem A. Mosa, Kundan Kumar, Sudesh Chhikara, Craig Musante, Jason C. White, Om Parkash Dhankher

**Affiliations:** 1 Stockbridge School of Agriculture, University of Massachusetts, Amherst, MA 01003, USA; 2 Department of Biotechnology, Faculty of Agriculture, Al-Azhar University, Cairo, Egypt; 3 Department of Applied Biology, College of Sciences, University of Sharjah, P.O. Box 27272, Sharjah, United Arab Emirates; 4 Department of Biological Sciences, Birla Institute of Technology & Science, K. K. Birla Goa Campus, Goa 403726, India; 5 Department of Analytical Chemistry, The Connecticut Agricultural Experiment Station, New Haven, CT 06504-1106, USA

## Abstract

High boron (B) concentration is toxic to plants that limit plant productivity. Recent studies have shown the involvement of the members of major intrinsic protein (MIP) family in controlling B transport. Here, we have provided experimental evidences showing the bidirectional transport activity of rice OsPIP1;3 and OsPIP2;6. Boron transport ability of OsPIP1;3 and OsPIP2;6 were displayed in yeast HD9 mutant strain (∆*fps1*∆*acr3*∆ycf1) as a result of increased B sensitivity, influx and accumulation by OsPIP1;3, and rapid efflux activity by OsPIP2;6. RT-PCR analysis showed strong upregulation of OsPIP1;3 and OsPIP2;6 transcripts in roots by B toxicity. Transgenic *Arabidopsis* lines overexpressing OsPIP1;3 and OsPIP2;6 exhibited enhanced tolerance to B toxicity. Furthermore, B concentration was significantly increased after 2 and 3 hours of tracer boron (^10^B) treatment. Interestingly, a rapid efflux of ^10^B from the roots of the transgenic plants was observed within 1 h of ^10^B treatment. Boron tolerance in OsPIP1;3 and OsPIP2;6 lines was inhibited by aquaporin inhibitors, silver nitrate and sodium azide. Our data proved that OsPIP1;3 and OsPIP2;6 are indeed involved in both influx and efflux of boron transport. Manipulation of these PIPs could be highly useful in improving B tolerance in crops grown in high B containing soils.

Boron (B) is an essential and immobile micronutrient required for all plant nutrition. The main functions of B relate to cell wall strength, nucleic acid synthesis, hormone responses, membrane function and cell cycle regulation^1^,^2^. Higher concentration of B is toxic to plants and leads to nutritional disorder that eventually limits plant production in arid and semi-arid environment^3^. High concentrations of B may occur naturally in soil or in groundwater, or added to the soil from mining, fertilizers, or irrigation water^4^. Recent studies have implicated the involvement of efflux type B transporters and members of major intrinsic protein (MIP) family in controlling B toxicity tolerance^5^.

Major Intrinsic Protein (MIP) superfamily is highly conserved with members ranging in size from 23 to 31 kDa^6^. In higher plants, MIPs are divided into five main subfamilies based on their sequence similarities and localization: Plasma membrane Intrinsic Proteins (PIPs), Tonoplast membrane Intrinsic Proteins (TIPs), Nodulin 26-like Intrinsic membrane Proteins (NIPs) and the Small basic Intrinsic Proteins (SIPs)^7^,^8^. Recently, uncharacterized X Intrinsic Proteins (XIPs) were identified in some plant and moss species^9^,^10^. Among the MIP subfamilies, members of the PIP subfamily are the most studied. PIP subfamily is further divided into two groups: PIP1s and PIP2s. PIP1s isoforms have very low water channel activity^11^, whereas, PIP2s isoforms have been shown to posses high water channel activity^12^,^13^. In rice, MIP family is comprised of 11 PIPs, 10 TIPs, 10 NIPs and two SIPs members^14^.

Several researchers have identified a number of B efflux transporters in plants. The first efflux-type B transporter identified was AtBOR1 from *Arabidopsis thaliana*^15^. Overexpression of AtBOR1 conferred tolerance to *Arabidopsis* under B deficient conditions and plays a key role in xylem loading^16^. BOR1 homolog in barley (*Hordeum vulgare*), HvBot1, has been identified as tolerance QTL encoding a putative transmembrane B transporter with similarity to bicarbonate transporter in animals^17^, enabled the barley plant to tolerate high levels of B^18^. Overexpression of an AtBOR1 paralog, AtBOR4, in transgenic *Arabidopsis* plants also increased their tolerance to high B levels^19^. Homologues of AtBOR1, *Hv-BOR2* and *Ta-BOR2* from barley and wheat, respectively, were cloned and positive correlations between mRNA levels of *BOR2* genes and tolerance of high B were described among different cultivars in both barley and wheat, supporting the role of BOR2 in tolerance of high B^18^. AtBOR2, encodes an efflux B transporter in *Arabidopsis* which is localized in plasma membrane, and is strongly expressed in lateral root caps and epidermis of elongation zones of roots and have role in crosslinking of rhamnogalacturonan II and root elongation under boron limitation in *Arabidopsis. BOR2* and *BOR1* mutants had reduced root elongation under low B availability^20^. In rice, OsBOR4, a boron efflux transporter, is required for normal pollen germination and/or pollen tube elongation, and homozygous mutants showed defects in pollen tube germination and/or elongation^21^.

Among MIPs, AtNIP5;1 was the first B transporter gene identified from *Arabidopsis* which is required for efficient uptake of B in *Arabidopsis* roots^22^. *Arabidopsis* NIP6;1 functions in xylem–phloem transport for preferential distribution of B into young growing tissues^23^. In barley, HvNIP2;1, was proposed for B toxicity tolerance, and mediated by reduced expression of HvNIP2;1 to limit B uptake^24^. A homolog of AtNIP5;1 was identified as MtNIP3 from *Meidicago truncatula* and proposed to be associated with B tolerance^25^. An MIP subfamily member, AtTIP5;1 has been shown to be responsible for providing tolerance to B toxicity in overexpressed transgenic *Arabidopsis* plants^26^. PIPs subfamily has been demonstrated to have a role in B permeability. The expresssion of maize Zm-PIP1 in *Xenopus laevis* oocytes resulted in increased B permeability^27^ and expression of Hv-PIP1;3 and Hv-PIP1;4 from barley increased the sensitivity of yeast cells to B^28^. Recently we reported that members of rice PIP2 subgroup, OsPIP2;4 and OsPIP2;7, have been shown to be involved in mediating B permeability and provide tolerance in overexpressed *Arabidopsis* plants most likely by internal redistribution of toxic B^29^.

In this present study, here we report the bidirectional transport activity of two rice PIPs; OsPIP1;3 and OsPIP2;6 as a representative members of PIP1 and PIP2 subgroups, respectively. Enhanced boron tolerance in the transgenic *Arabidopsis* plants overexpressing OsPIP1;3 and OsPIP2;6 is discussed.

## Results

### OsPIP1;3 Expression Enhances B Sensitivity and Increases B Content in Yeast

**Figure 1:**
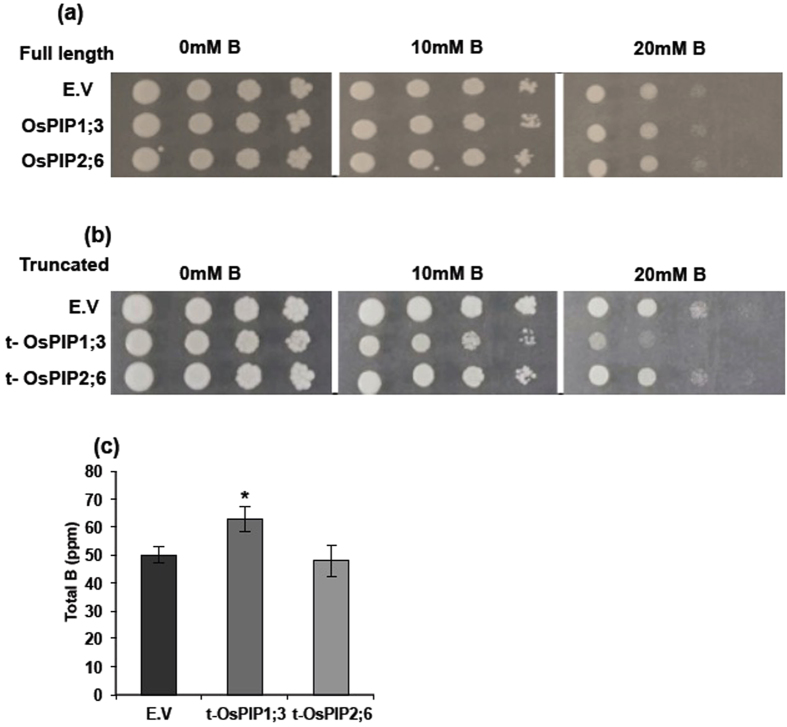
Functional complementation of fps1 for B transport by OsPIP1;3 and OsPIP2;6 in *S. cerevisiae* HD9 strain (∆*fps1*∆*acr3*∆*ycf1*). Expression of full length (**a**) and truncated version (**b**) of OsPIP1;3 and OsPIP2;6 in HD9 yeast strain. The transformants were grown in liquid medium and 10-fold serial dilutions of the culture were spotted on plates with boric acid. Growth was recorded after 3–4 days at 30 °C. (**c**) Boron accumulation in yeast cells expressing pYES3 empty vector, t-OsPIP1;3, and t-OsPIP2;6. Data are means ± S.D (n = 3), *P < 0.05.

We tested the ability of OsPIP1;3 and OsPIP2;6, as a representative members of PIP1 and PIP2 subgroups, to functionally complement the function of aquaglyceroporin Fps1 in *S. cerevisae*. Both *OsPIP1;3*, and *OsPIP2;6* genes were expressed in the HD9 yeast strain (∆*fps1*∆*acr3*∆*ycf1*) lacking the expression of the bidirectional aquaglyceroporin pump Fps1, arsenite export pump ACR3, and the vacuolar transporter YCF1^30^, for complementation of the B sensitivity and mobilization. The preliminary results with full-length coding region of OsPIPs failed to show any B transport activity in yeast HD9 strain. There was no difference in the growth of HD9 strain (∆*fps1*∆*acr3*∆*ycf1*) expressing OsPIP genes and the empty vector controls (Fig. 1A). It has been reported that some of the plant aquaporins required a truncation of the N-terminal hydrophobic region in order to complement the function in yeast Fps1 mutant^31^. Therefore, we attempted to complement the B transport activity of OsPIP1;3, and OsPIP2;6 after truncating their N-terminal hydrophobic region. Yeast growth was strongly impaired in cells expressing truncated OsPIP1;3 compared to yeast transformed with the pYES3 empty vector control on plates containing medium supplemented with 10 mM and 20 mM of boric acid (Fig. 1B). However, there was no significant difference between pYES3 empty vector and yeast expressing OsPIP2;6.

Total B content in the yeast cells expressing the truncated OsPIP1;3 and OsPIP2;6 was analyzed. The yeast cells expressing OsPIP1;3 showed significantly higher B accumulation as compared with pYES3 vector control (Fig. 1C), whereas, yeast cells expressing OsPIP2;6 had no significant difference in the B accumulation level compared to yeast cells expressing pYES3 empty vector. This B accumulation data is in accordance with the B sensitivity phenotype in HD9 strain complemented with OsPIP1;3.

### Short Duration Influx and Effux of B in HD9 Yeast Strain Expressing OsPIP1;3 and OsPIP2;6

The role of OsPIP1;3 and OsPIP2;6 in transporting B was evaluated by measuring short duration influx assay using tracer ^10^B. HD9 strain expressing the empty vector pYES3, OsPIP1;3, and OsPIP2;6 were grown to an OD_600_ of 1. For influx, cells were exposed to tracer boron ^10^B enriched boric acid for 0, 15, 30 and 60 min and ^10^B contents were measured by ICP-MS. Expression of OsPIP1;3 led to a significant increase in ^10^B uptake after 15, 30 and 60 min of exposure. While, there was no significant difference between yeast cells expressing the empty vector pYES3 and OsPIP2;6 (Supplementary Fig S1A). This time dependent influx results corresponded well with the levels of B sensitivity observed when growing the respective transformants on B-containing medium as shown in Fig. 1A.

To test whether OsPIP1;3 and OsPIP2;6 have B efflux activity, HD9 strains expressing the empty vector pYES3 and truncated versions of OsPIP1;3 and OsPIP2;6 were allowed to accumulate ^10^B till the end of log phase, harvested, washed, and then cells were re-suspended in B free media. ^10^B contents were analyzed by the method previously described after 15, 30 and 60 minutes. Efflux from yeast cells expressing OsPIP2;6 was faster than yeast cells expressing the empty vector pYES3 and OsPIP1;3 after 15 minutes of resuspension in B free media. However, there was no difference between the empty vector pYES3 and OsPIP2;6 after 30 and 60 minutes of resuspension in B free media. Whereas, at all time points, yeast cells expressing OsPIP1;3 showed less efflux activity than the empty vector pYES3 and OsPIP2;6 (Supplementary Fig. 1B).

### Differential Regulation of OsPIP1;3 and OsPIP2;6 Under Boron Toxicity

**Figure 2:**
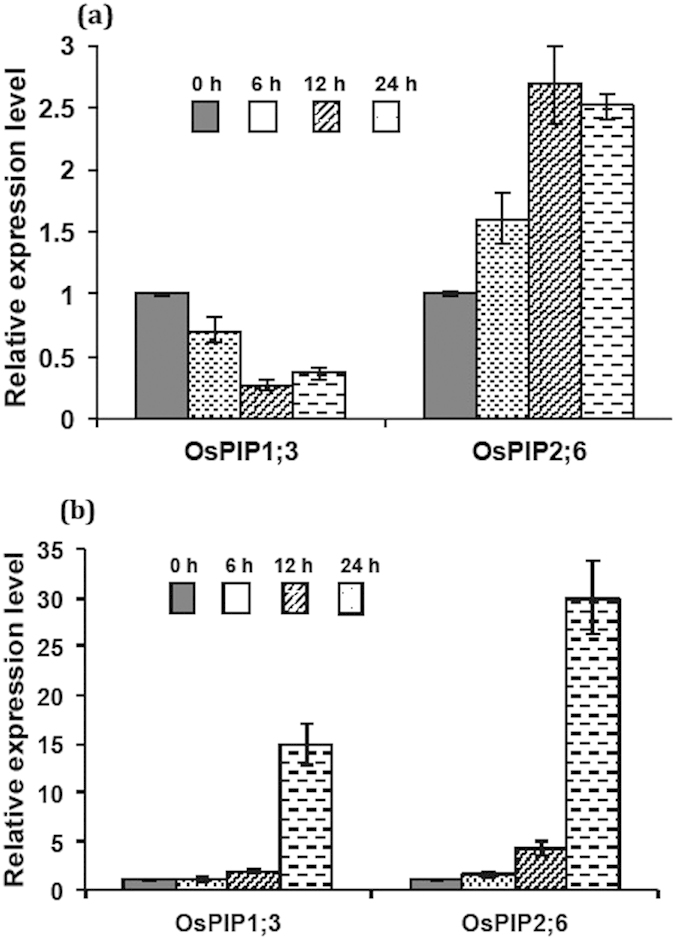
Relative expression of OsPIP1;3 and OsPIP2;6 genes in (**a**) shoots and (**b**) roots in response to boron exposure. Rice seedlings were exposed to 5 mM boric acid before harvesting shoots and roots tissues after 0, 6, 12 and 24 hrs of boron treatment. 18S rRNA was used for normalization of gene expression.

To evaluate the differential expression of *OsPIP1;3* and *OsPIP2;6 *mRNA transcripts in rice root and shoot tissues under B toxicity, we performed a quantitative RT-PCR (qRT-PCR) analysis. In shoots, transcript level of *OsPIP1;3* were downregulated after 6, 12, and 24 hrs of B treatment, whereas, *OsPIP2;6* transcript levels showed contrasting pattern as it was upregulated after 6, 12 and 24 hrs of B toxicity (Fig. 2A). In roots, our results showed that the transcript levels of *OsPIP1;3* and *OsPIP2;6* were strongly upregulated up to 24 hrs of B treatment compared to the untreated controls (Fig. 2B). The transcript level for *OsPIP1;3* showed 15-fold increase at 24 hrs of B exposure, whereas, *OsPIP2;6* were induced by 30-fold at 24 hrs of B toxicity (Fig. 2B).

### Enhanced Boron Toxicity Tolerance in Arabidopsis Plants Overexpressing OsPIP1;3 and OsPIP2;6

To investigate the function of OsPIP1;3 and OsPIP2;6 in boron permeability in plants, OsPIP1;3 was overexpressed in *A. thaliana* under *actin2* promoter-terminator cassette (*ACT2pt*) (Supplemental Fig. S2A). Following transformation, several independent transgenic lines were screened for kanamycin resistance, and then three T_2_ homozygous *Arabidopsis* lines overexpressing OsPIP1;3 (43, 51, and 60) were selected for further analysis. In addition, we used the three T_2_ homozygous *Arabidopsis* lines overexpressing OsPIP2;6 (27, 33, and 40) that we generated in our previous study^32^ for further analysis. The overexpression of each transgene in the transgenic T_2_ *Arabidopsis* plants was confirmed by semi-quantitative RT-PCR analysis (supplementary Fig. S2B).

**Figure 3:**
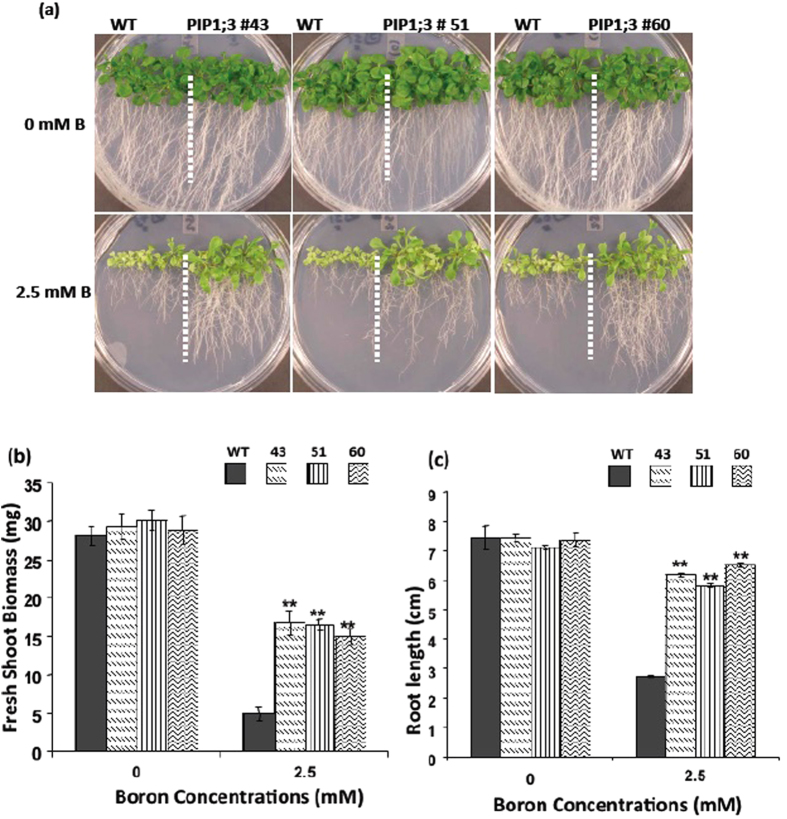
Boron resistance phenotype of transgenic *Arabidopsis* overexpressing OsPIP1;3. (**a**) Boron tolerance phenotype, (**b**) Fresh shoot weight and, (**c**) root length of the *Arabidopsis* transgenic lines 43, 51 and 60 as compared with wild type (WT) on 0 and 2.5 mM boric acid. The values are presented as an average of four replicates of 40 plants. The asterisks represent the significant difference in biomass accumulation and root length compared with wild type (WT) plants, *P < 0.05, **P < 0.01.

**Figure 4:**
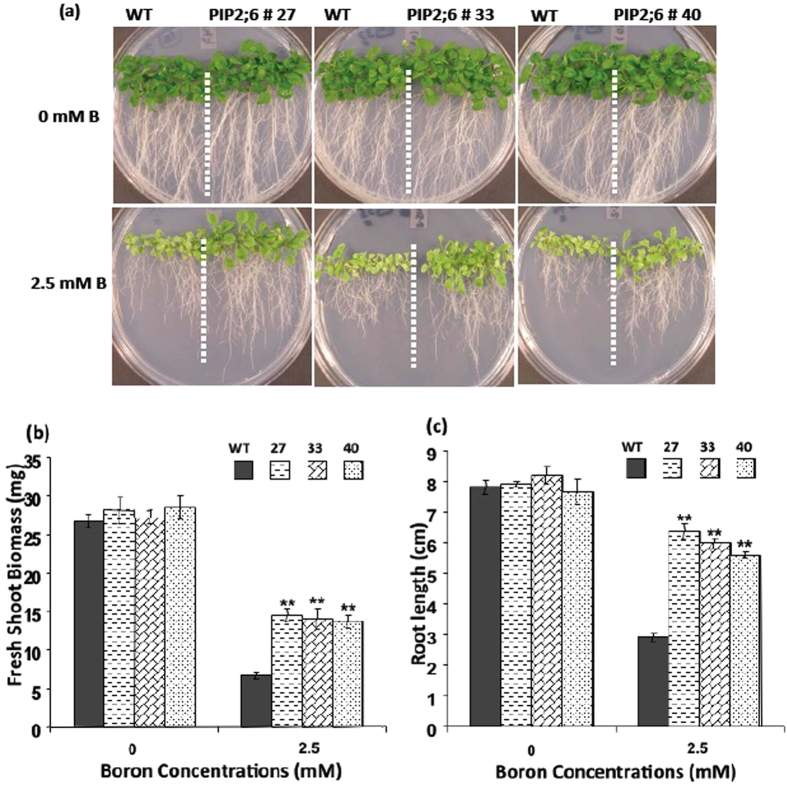
Boron resistance phenotype of transgenic *Arabidopsis* overexpressing OsPIP2;6. (**a**) Boron tolerance phenotype, (**b**) Fresh shoot weight and, (**c**) root length of the *Arabidopsis* transgenic lines 27, 33 and 40 as compared with wild type (WT) on 0 and 2.5 mM boric acid. The values are presented as an average of four replicates of 40 plants. The asterisks represent the significant difference in biomass accumulation and root length compared with wild type (WT) plants, *P < 0.05, **P < 0.01.

To evaluate the phenotypic effect of B on the *Arabidopsis* transgenic lines overexpressing OsPIP1;3 and OsPIP2;6, seeds of transgenic lines and wild-type *Arabidopsis* were germinated on an 1/2x MS agar medium containing 0 or 2.5 mM of boric acid. On the control media without B, there were no differences in the phenotypes between the transgenic lines overexpressing OsPIP1;3 and OsPIP2;6, and wild-type control plants. However, on media supplemented with 2.5 mM of boric acid, the transgenic lines exhibited strong tolerance to B toxicity as compared with wild type plants (Figs 3 and 4). The growth of both shoots and roots of the transgenic lines were more vigorous than that of wild type plants on media containing B. The OsPIP1;3 and OsPIP2;6 transgenic plants had well developed green leaves compared to the wild types that had smaller and pale yellow leaves. Further, OsPIP1;3 and OsPIP2;6 transgenic plants demonstrated well-branched and longer roots compared to wild type plants (Figs 3A and 4A). The fresh shoot weight of OsPIP1;3 and OsPIP2;6 transgenic lines was significantly higher as compared to wild type plants on media supplemented with 2.5 mM B (Figs 3B and 4B). The root length of OsPIP1;3 and OsPIP2;6 overexpression lines were more than two-fold longer than that of the wild type plants on medium containing 2.5 mM B (Figs 3C and 4C).

### Boron Accumulation in Transgenic Arabidopsis Overexpressing Rice PIP1;3 and PIP2;6

**Figure 5:**
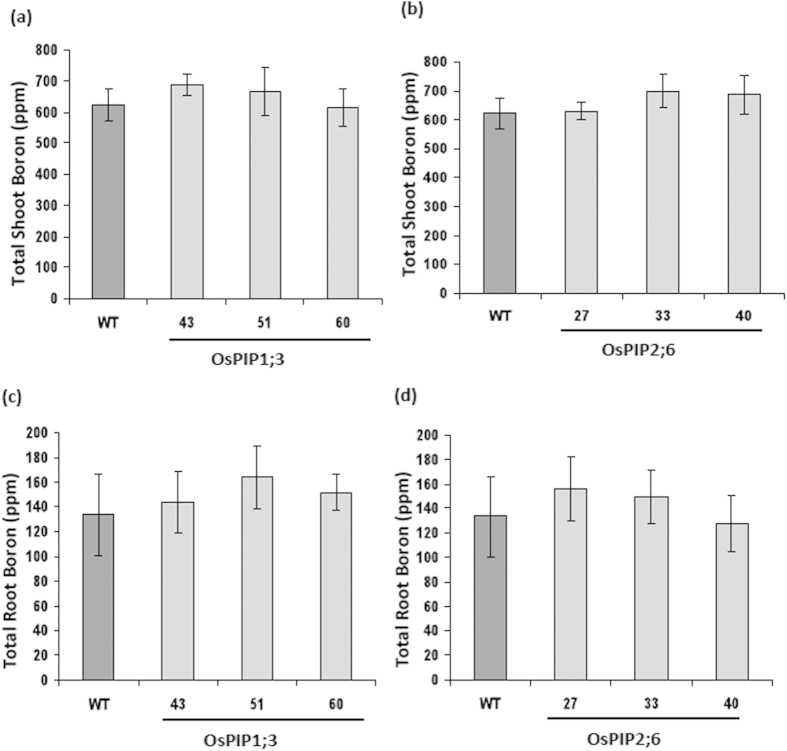
Analysis of total B accumulation in the transgenic Arabidopsis lines expressing OsPIP1;3 and OsPIP2;6. Total shoot B (**a**) in the transgenic *Arabidopsis* lines 43, 51, and 60 overexpressing OsPIP1;3 and (**b**) in the transgenic *Arabidopsis* lines 27, 33 and 40 overexpressing OsPIP2;6 in comparison with wild type (WT). Total root B (**c**) in the transgenic *Arabidopsis* lines 43, 51 and 60 overexpressing OsPIP1;3 and (**d**) in the transgenic *Arabidopsis* lines 27, 33 and 40 overexpressing OsPIP2;6 in comparison with wild type (WT). The average and standard error (SE) values are shown for four replicates of 25 plants each for WT and transgenic *Arabidopsis* lines.

To test whether OsPIP1;3 and OsPIP2;6 are involved in B transport and uptake, wild type and transgenic *Arabidopsis* lines overexpressing OsPIP1;3 and OsPIP2;6 were grown hydroponically for three weeks and then treated with 2.5 mM boric acid for four days. Total B from shoots and roots of wild type and transgenic *Arabidopsis* were measured separately by ICP-MS. In shoots, overexpression of OsPIP1;3 and OsPIP2;6 showed no significant difference in B accumulation compared to the wild type (Fig. 5A,B). Similarly, there were no significant differences in B accumulation levels between the transgenic lines overexpressing OsPIP1;3 and OsPIP2;6 root tissues compared to wild type plant roots (Fig. 5C,D).

To confirm these results, another long-term uptake assays were conducted using a stable isotope of B (^10^B). Regular Boric acid composition is ^11^B : ^10^B = 81.0 : 19.0. Wild type and transgenic *Arabidopsis* lines overexpressing OsPIP1;3 and OsPIP2;6 were grown on 1/2x MS for three weeks and then treated with 2.5 mM^10^B enriched boric acid for four days. Accumulation of ^10^B in shoots and roots of wild type and transgenic plants were measured separately by ICP-MS. Similar to previous results for boric acid, there was no significant difference in ^10^B accumulation levels between the *Arabidopsis* wild type plants and the transgenic lines overexpressing OsPIP1;3 and OsPIP2;6 in both shoot and root tissues (Supplemental Fig. S3).

### OsPIP1;3 and OsPIP2;6 Exhibited Bidirectional Transport Activity in Transgenic Arabidopsis Plants

**Figure 6:**
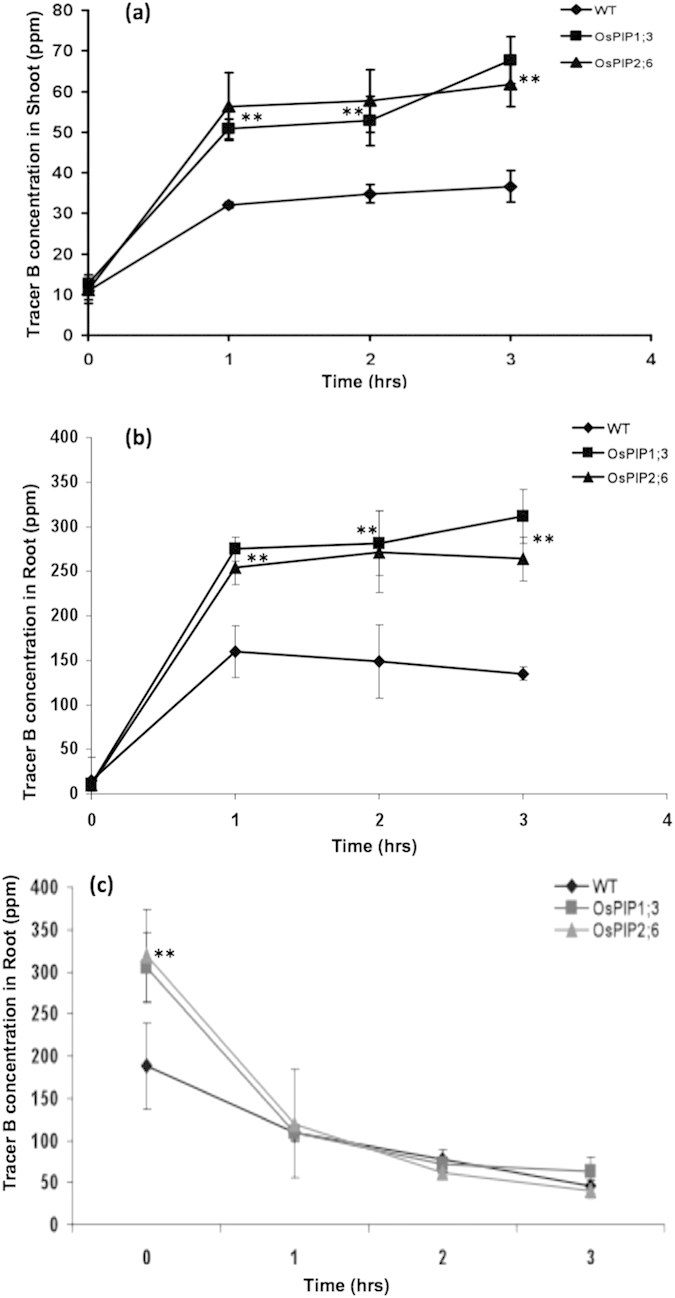
Influx and efflux of boron in the transgenic *Arabidopsis* lines expressing OsPIP1;3 and OsPIP2;6. Concentration of tracer boron ^10^B in shoot (**a**) and roots (**b**) of transgenic *Arabidopsis* expressing OsPIP1;3 and OsPIP2;6 in comparison with wild type (WT) during short-term exposure to 2.5 mM ^10^B enriched boric acid for 1, 2, and 3 hrs. (**c**) Concentration of tracer boron ^10^B in roots of transgenic *Arabidopsis* expressing OsPIP1;3 and OsPIP2;6 in comparison with wild type (WT) during short-term efflux of 2.5 mM ^10^B enriched boric acid for 1, 2, and 3 hrs. The average and standard error (SE) values are presented for three replicates of 25 plants each for WT and transgenic *Arabidopsis* lines. The asterisks represent the significant difference in influx and efflux of B compared with wild type (WT) plants, *P < 0.05, **P < 0.01.

In order to check if OsPIP1;3 and OsPIP2;6 are involved in the influx of B, a short-term B uptake (influx) using a stable isotope ^10^B was performed. Wild type and transgenic *Arabidopsis* plants overexpressing OsPIP1;3 and OsPIP2;6 were first grown on 1/2x MS containing required amount of boric acid (^11^B : ^10^B = 81.0 : 19.0) for 3 weeks, and then plants were exposed to media containing 5 mM ^10^B enriched boric acid and the amount of ^10^B taken up by roots and shoots were determined at specified time points. In shoots, OsPIP1;3 and OsPIP2;6 overexpressed plants exhibited greater ^10^B uptake than wild type plants after 1, 2, and 3 hrs (Fig. 6A). Similarly, a rapid influx of ^10^B across the roots of OsPIP1;3 and OsPIP2;6 transgenic plants after 1, 2 and 3 hrs of exposure was observed (Fig. 6B).

To determine whether OsPIP1;3 and OsPIP2;6 are involved in the efflux of B, a short-term B efflux assay using a stable isotope ^10^B was conducted. *Arabidopsis* wild type and transgenic plants overexpressing OsPIP1;3 and OsPIP2;6 were first exposed to 5 mM ^10^B enriched boric acid in 1/2x MS liquid medium for 3 hrs and then incubated in 1/2x MS liquid medium without any B up to 3 hrs. Concentration of ^10^B in roots was measured at each time point (Fig. 6C). A rapid efflux of more than half of ^10^B was observed within the first hour after transferring to 1/2x MS media without B in roots of the transgenic *Arabidopsis* expressing OsPIP1;3 or OsPIP2;6. However, after 2 and 3 hours period there was no significant difference in ^10^B concentration in roots of the transgenic and wild types control plants (Fig. 6C).

### Effect of Aquaporin Inhibitors on Boron Tolerance in OsPIP1;3 and OsPIP2;6 Transgenic Plants

**Figure 7:**
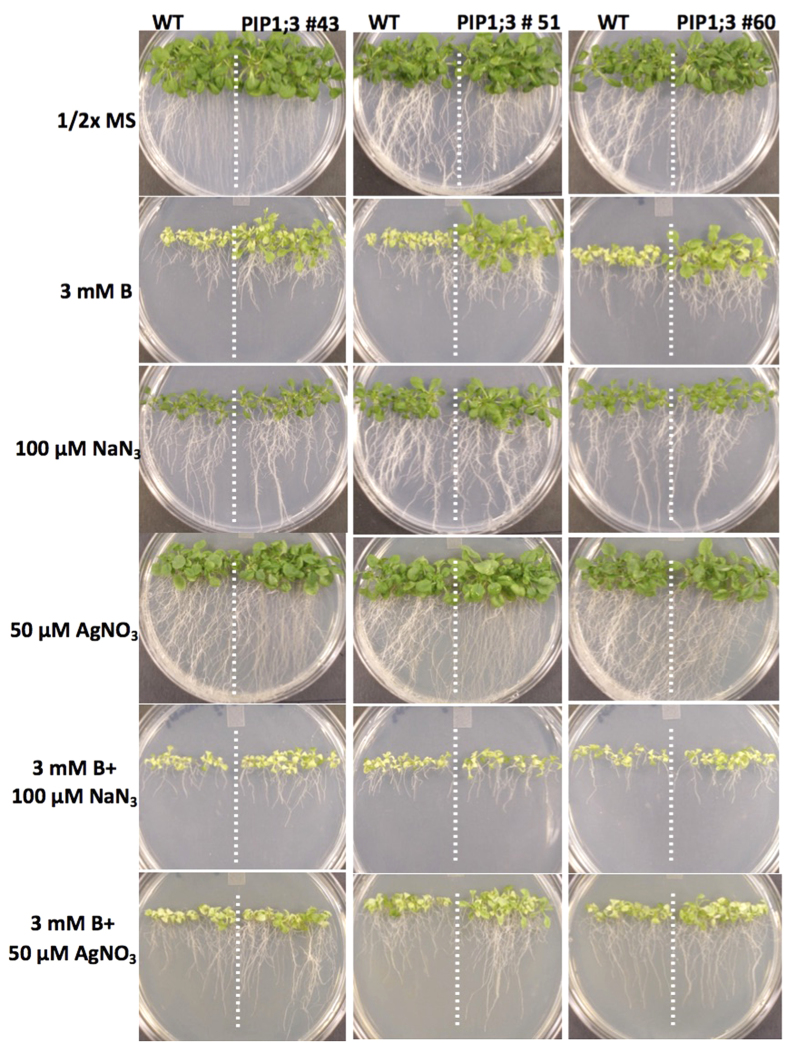
Phenotype of *Arabidopsis* expressing OsPIP1;3 on boron containing media and boron transporter inhibitors. *Arabidopsis* transgenic lines 43, 51 and 60 expressing OsPIP1;3 as compared with wild type (WT) on 0, 3 mM B, 100 μM sodium azide, 50 μM silver nitrate, 3 mM B + 100 μM sodium azide, and 3 mM B + 50 μM silver nitrate.

**Figure 8:**
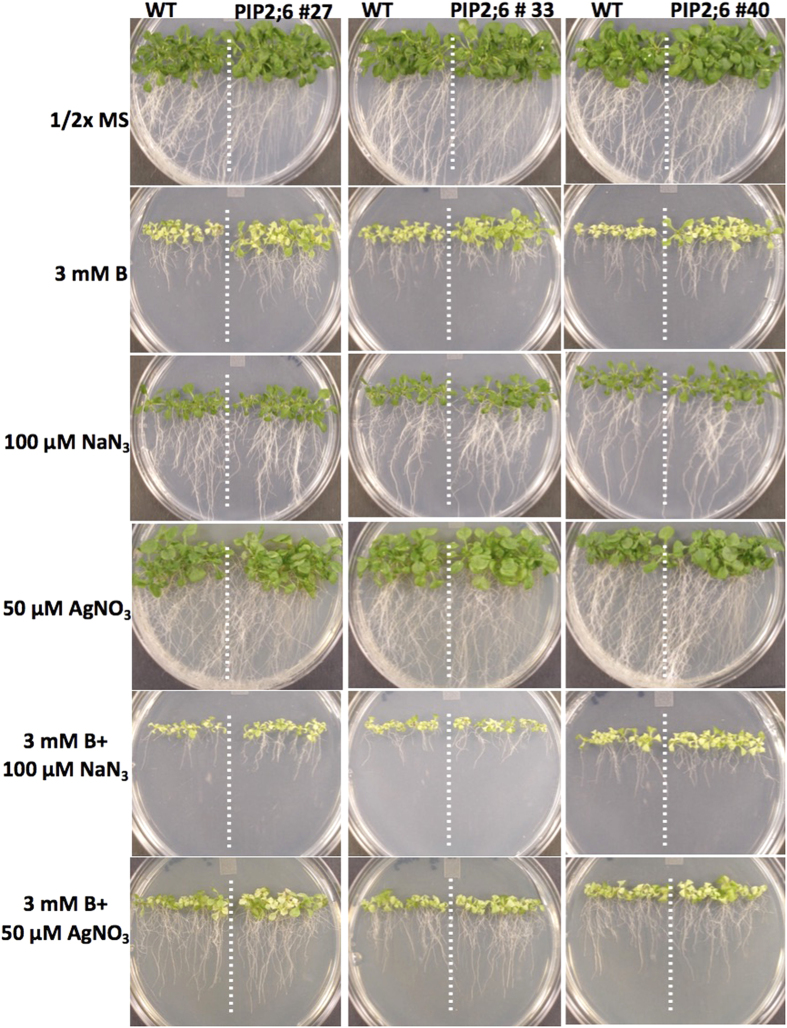
Phenotype of *Arabidopsis* expressing OsPIP2;6 on boron containing media and boron transporter inhibitors. *Arabidopsis* transgenic lines 27, 33 and 40 expressing OsPIP2;6 as compared with wild type (WT) on 0, 3 mM B, 100 μM sodium azide, 50 μM silver nitrate, 3 mM B + 100 μM sodium azide, and 3 mM B + 50 μM silver nitrate.

These above-described findings prompted further investigation into the effect of two different aquaporin channel inhibitor/blocker, sodium azide (NaN_3_) and silver nitrate (AgNO_3_), on the phenotype of the *Arabidopsis* plants overexpressing OsPIPs. Seeds of *Arabidopsis* wild type control and three independent transgenic lines were grown for three weeks on 1/2x MS medium containing 0, 3 mM B, 100 μM NaN_3_, 50 μM AgNO_3_, 3 mM B + 100 μM NaN_3_, and 3 mM B + 50 μM AgNO_3_. There was no difference between the wild type plants and transgenic lines overexpressing OsPIP1;3 and OsPIP2;6 grown on the control plates without B or NaN_3_ or AgNO_3_ (Figs 7 and 8). However, as expected, the transgenic lines of OsPIP1;3 and OsPIP2;6 grown on 3 mM B were more tolerant than the wild type plants. Interestingly, when plants were grown on media containing 3 mM B + 100 μM NaN_3_, tolerance to B was abolished and there were no phenotypic differences between transgenic OsPIP1;3, OsPIP2;6 lines and wild-type control plants. Similarly, the addition of 50 μM AgNO_3_ to the 3 mM B media decreased the tolerance phenotype of the transgenic lines overexpressing OsPIP1;3 and OsPIP2;6 (Figs 7 and 8). The fresh shoot biomass of the OsPIP1;3 and OsPIP2;6 transgenic lines had 2 to 3-fold more the biomass of wild type controls when grown on 3 mM B (Supplementary Fig. S4). Whereas, the biomass of the OsPIP1;3 and OsPIP2;6 transgenic lines and wild type plants was almost same when plants were grown on 3 mM B + 100 μM NaN_3_ or 3 mM B + 50 μM AgNO_3_ (Supplementary Fig. S4). This proves the specific action of the inhibition of PIPs by sodium azide and silver nitrate and the tolerance to B in the transgenic lines is as a result of the activity of the OsPIP1;3 and OsPIP2;6.

## Discussion

Aquaporins are shown to play a pivotal role in metalloids transport in various organisms and our previous reports potentiate the role of members of rice PIPs, a subfamily of aquaporins in arsenite (AsIII) and B transport in plants^32^,^29^. In current study, we have provided experimental evidences showing that OsPIP1;3 and OsPIP2;6 are involved in both influx and efflux of boron transport. Regulation analysis of *OsPIP1;3* and *OsPIP2;6* transcripts showed that both genes exhibited a strong upregulation in roots under B toxicity. The reason for slight decreases in OsPIP1;3 transcript levels in shoots compared to OsPIP2;6 is not known. However, given to the very high levels of transcripts of both OsPIP1;3 and OsPIP2;6 in roots (15- to 30-fold higher), this difference (2-fold decrease) in shoot is relatively minor. Further, this difference in the regulation of both genes in shoots might be due their difference in tissue-specificity or functional variability, which need further studies to clarify. It has previously been reported that expression of HvPIP1;3 boron transporter was significantly increased upon exposure to 5 mM of B in barley^28^. Similarly, the expression of OsPIP2;4 and OsPIP2;7 were strongly induced in roots by toxic B treatment, that favors the current evidences^29^.

The ability of OsPIP1;3 and OsPIP2;6 to transport B was examined using a yeast complementation assay with HD9 strain defective in Fps1, ACR3, and Ycf1^30^. Preliminary results with full-length genes failed to show any B transport activity in yeast strain HD9. It has been reported that some members of plant PIPs require partial truncation of the hydrophobic N-terminal domains in order to be functionally expressed in yeast^28^,^29^. Therefore, in the current study, we attempted to complement the B transport activity of OsPIP1;3 and OsPIP2;6 after truncating their N-terminal hydrophobic region. The B transport ability of OsPIP1;3 could be demonstrated as a result of increased B sensitivity and increased B influx and accumulation in yeast cells. Whereas, there was no difference in the growth of HD9 strain expressing OsPIP2;6 and the empty vector control. However, our observation should not be directly taken as the inability of OsPIP2;6 to transport boron as it showed strong efflux activity of B to external media within 15 minutes. This rapid efflux explains the B-insensitive phenotype and lack of B accumulation in yeast strain. Similarly, earlier studies also showed that expression of OsNIP1;1 did not affect the growth of Δ*fps1*Δ*acr3*Δ*ycf1* mutant yeast on AsIII containing medium^31^. However, OsNIP1;1 was able to mediate AsIII transport when expressed in *Xenopus* oocytes^33^. In addition, heterologous expression of NIP1;2, NIP5;1, NIP6;1, NIP7;1 in yeast demonstrated their ability to transport AsIII^31^,^34^, although AsIII tolerance was not observed in the T-DNA lines of *nip1;2, nip5;1, nip6;1*, and *nip7;1*^35^. Therefore, these inconsistent results may rely on the respective expression assays and experimental conditions. Likewise, considering the fact that the failure of complementation of OsPIP2;6 in HD9 strain, our study also demonstrated the B transport ability of OsPIP2;6 in the overexpressed *Arabidopsis* transgenic plants. Further, we stressed that yeast cells are different than plant cells in terms of their ability to tolerate B toxicity. Optimization of yeast and *Arabidopsis* growth on various concentrations of boric acids showed clearly that yeast can tolerate much higher (up to 20 mM) concentration of B than Arabidopsis (up to 3 mM).

When OsPIP1;3 and OsPIP2;6 were overexpressed in *Arabidopsis* to clarify their physiological function *in planta*, the transgenic plants exhibited enhanced tolerance to B toxicity. Similar results were previously observed for other aquaporins members. Transgenic *Arabidopsis* plants overexpressing AtTIP5;1 conferred tolerance to B toxicity^26^. Overexpression of OsPIP2;4 and OsPIP2;7 in *Arabidopsis* also resulted in an increased tolerance to toxic B levels^29^. It is also intriguing that total B concentration was not affected when the transgenic *Arabidopsis* plants overexpressing OsPIP1;3 and OsPIP2;6 treated with boric acid for four days while the B concentration was significantly increased after 2 and 3 hours of ^10^B treatment. Interestingly, a rapid efflux of ^10^B from the roots of the OsPIP1;3 and OsPIP2;6 overexpressed transgenic *Arabidopsis* plants was observed within 1 h of ^10^B treatment. These results are in accordance with our recent report demonstrated the involvement OsPIP2;4 and OsPIP2;7 in both influx and efflux of B from roots and shoot tissues^29^, which is also consistent with the well-known fact of the metalloids bidirectional transport properties of plant aquaporins^31^. Therefore, tolerance to toxic B in the OsPIP1;3 and OsPIP2;6 overexpressed transgenic *Arabidopsis* plants presumed to be as a result of the rapid efflux of the excess B from root tissues to external medium.

Further, it is interesting to note that this B tolerance phenotype in OsPIP1;3 and OsPIP2;6 overexpressed lines caused by efflux mechanism was inhibited by two aquaporin inhibitors; silver nitrate and sodium azide. It has been reported that silver nitrate and sodium azide can block the aquaporins channels in plants^36^,^37^. Arsenite uptake in roots and fronds of *P. Vittata* was reduced by 64% and 58% in response to silver nitrate treatment as a possible result of inhibiting aquaporin transporters^38^. Boron transport via PIP aquaporins in barley roots was reduced by 40–50% in response to metabolic inhibition by sodium azide^28^. In this study, we demonstrated phenotypic evidence showing the inhibition of aquaporin gating by silver nitrate and sodium azide. Adding these two inhibitors to the media containing toxic B, abolished the tolerance phenotype of the transgenic *Arabidopsis* plants overexpressing OsPIP1;3 and OsPIP2;6 as a result of blocking the transport (both influx and efflux) mechanism of PIPs.

**Figure 9:**
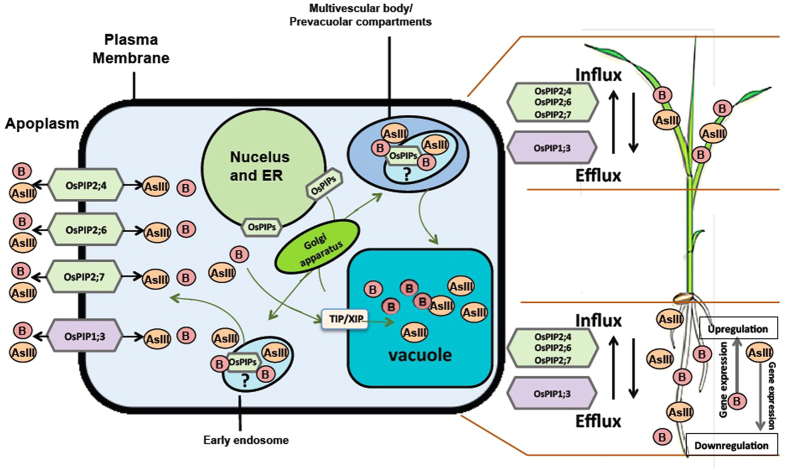
Schematic diagram indicating boron and arsenite (AsIII) transport mechanisms through OsPIPs and their differential regulation by boron and AsIII. The bidirectional transport (influx and efflux) through OsPIP1;3, OsPIP2;4, OsPIP2;6 and OsPIP2;7 proteins localized at the plasma membrane have been demonstrated in the diagram. Other possible unconfirmed mechanisms (marked with ‘?’) is represented here, PIPs might be involved in the internal re-distribution of metalloids by transporting it from the endoplasmic reticulum (ER) via the Golgi apparatus to the plasma membrane. Also, metalloids could be transported through early endosome to the multivescular body/prevacuolar compartments before it’s finally placed into vacuole. AsIII and B may be transported to vacuole by uncharacterized X Intrinsic Proteins (XIPs) and/or the Tonoplast membrane Intrinsic Proteins (TIPs).

In conclusion, the findings presented here suggesting a bidirectional B transport activity of OsPIP1;3 and OsPIP2;6 and their differential regulation in roots and shoots exposed to high B. These results are in agreement with our previous findings for OsPIP2;4 and OsPIP2;7^29^, supporting our hypothesis that PIPs are involved in providing B tolerance by maintaining the B homeostasis in plant cells via their bidirectional transport activity under elevated B stress conditions. Similarly, we have previously shown the overexpression of OsPIP2;4, OsPIP2;6 and OsPIP2;7 showed strong tolerance to AsIII via similar bidirectional transport activity and were differentially regulated by AsIII^32^. Based on these findings, we drew a working model (Fig. 9) showing a proposed mechanism along with conclusion that for OsPIP1;3, OsPIP2;4, OsPIP2;6 and OsPIP2;7, contribute largely to the B and AsIII transport and tolerance mechanism in plants. OsPIP1;3, OsPIP2;4, OsPIP2;6, and OsPIP2;7 proteins localized at the plasma membrane are involved in the bidirectional transport of AsIII and B, and most likely other metalloids as well, which need to be tested in the future studies. More studies are needed to investigate the other possible mechanisms marked with ‘?’ in Fig. 9. PIPs could transport metalloids through early endosome to vacuole or to the plasma membrane, supporting the hypothesis that it might be involved in the internal re-distribution of metalloids (Fig. 9). Further characterization of these and other PIPs is required to clarify the functional significance of each member in boron transport in plants. Manipulation of these PIPs in crops could prove highly useful in improving B tolerance in crops grown in high B containing soils.

## Methods

### Plant Material and Boron Treatment

Rice (*Oryza sativa* cv. Nipponbare) seeds were germinated for two weeks in vermiculite under the green house conditions of 16/8 hours light/dark cycle, respectively, at 28 °C. Two weeks old rice plants were transferred to hydroponic solution with Hoagland’s nutrients and kept to acclimatize for one week before the treatment. Five mM boric acid were added to hydroponic solution before harvesting shoots and roots tissues at 0, 6, 12 and 24 hrs after B treatments. Harvested tissues were frozen into liquid nitrogen, and stored at −80 °C until further use.

### RNA Extraction and RT-PCR Analysis

Total RNA from shoot and root tissues were isolated using RNeasy plant mini kit (Qiagen) following the manufacturer’s instructions. Five μg of total RNA was used for reverse transcription using the ThermoScript™ RT-PCR System (Invitrogen) for first-strand cDNA synthesis following the manufacturer’s instructions. Gene-specific primers used for the quantitative real time PCR (qRT-PCR) analysis were designed using the PrimerQuest (Intergrated DNA Technologies). The sequences of the qRT-PCR primers are as follows: OsPIP1;3 forward primer: CTGGTGATCGATGAAGCTAG; reverse primer: ACACAAGTACCATTTCTCACAC; and OsPIP2;6 forward primer: GCCAGGTGCATGATTTGTT; reverse primer: GCCGAAGCAGTTTGTATCTC. qRT-PCR was performed following the instruction for Mastercycler® ep realplex (Eppendorf AG, Hamburg, Germany) with ABsolute Blue qPCR SYBR Green Mix (Thermo Fisher Scientific, Surrey, UK). Relative expression level was calculated using 2^−ΔΔCT^ method^39^. Rice 18S ribosomal RNA (rRNA) was used as housekeeping genes for normalization. For semi-quantitative RT-PCR, Taq Polymerase (TaKaRa) was used according to the manufacturer’s instruction. The PCR amplification conditions were: 95 °C for 2 min; 95 °C for 45 s, 58 °C for 45 s, 72 °C for 45 s; final extension at 72 °C for 7 min.

### Yeast Complementation Assays

Full-length or truncated *OsPIP1;3* and *OsPIP2;6* gene sequences were amplified by PCR. For N-terminal truncation of OsPIPs, first 30–40 amino acids, were deleted by designing forward primer lacking these N-terminal sequences as previously described^29^. Amplified products were cloned into the pGEM-T easy vector (Promega, UK) using manufacturer’s instructions. The sequences were verified by DNA sequencing and then were cloned into *Eco*RI and *Xho*I sites of pYES3 vector for subsequent transformation into HD9 (∆fps1∆acr3∆ycf1) yeast (*Saccharomyces cerevisae*) strain^30^. The transformants were selected onto minimal SD medium minus tryptophan and confirmed by PCR. Yeast culture of HD9 expressing empty vector pYES3 or OsPIP1;3 and OsPIP2;6 were grown in liquid SD medium with either 2% glucose or 2% galactose and the appropriate supplements. Yeast phenotypes assays were performed as previously described by Kumar *et al*. (2013)^29^. Briefly, the cultures were diluted to an OD_600_ of 1.0. Cells with 10-fold dilutions series were spotted onto SD + Gal plates and SD + glu plates (as a control) with various concentrations of boric acid as indicated. The indicated concentration of boric acids were selected based on optimization of yeast strain growth conditions. Yeast growth on the plates was observed after 3–4 days of incubation at 30 °C.

### Boron Transport Assays in Yeast

HD9 strain transformed with OsPIP1;3 and OsPIP2;6 and empty vector pYES3 were grown at 30 °C in SD minus tryptophane medium supplemented with 2% galactose to an OD_600_ of 1.0. For B uptake assay, cells were supplied with 10 mM boric acid and allowed to grow overnight. Yeast cells were washed with Tris-HCl buffer containing 0.5% NaCl and then quickly washed by deionized water and dried at 70 °C, which were then digested with concentrated HNO_3_ and H_2_O_2_ for total B was analysis by ICP-MS (Perkin Elmer). For B influx assay, cells were exposed to 10 mM ^10^B enriched boric acid (99%, Cambridge Isotope Laboratories, Andover, MA, USA) for 0, 15, 30 and 60 min. Cells were harvested and washed once with chilled 25 mM phosphate buffer pH 6.0 containing 0.5% NaCl, then by 10 mM Tris HCl followed by deionized water and dried at 70 °C. Total B contents were estimated by ICP-MS (Perkin Elmer) after digestion with concentrated HNO_3_ and H_2_O_2_. For B efflux assay, cells were allowed to accumulate B till the end of log phase in 10 mM ^10^B enriched boric acid containing SD (-tryptophan) media. Cells were washed in chilled phosphate buffer as described above and re-suspended in the media without B. Aliquot of cells were withdrawn at 0, 20, 40 and 60 minute and the B content were analyzed by the method previously described. Total B contents at 20, 40 and 60 min were plotted as a fraction of B content at time 0, which is the maximum amount of B accumulated.

### Overexpression of OsPIP1;3 and OsPIP2;6 in Arabidopsis and Characterization of Transgenic Plants

Full-length fragments of *OsPIP1;3* gene was amplified by PCR using the following primers; Forward: TACGTCGAATTCTCCATGGAGGGGAAGGAGGA; Reverse: TAGCTGCTCGAGTTAGTCCCGGCTCTTGAA, and then cloned into the pGEMT easy vector (Promega, UK) using manufacturer’s instructions. The *Nco*I*/Xho*I fragment of *OsPIP1;3* was sub-cloned under the constitutively expressed *actin2* gene promoter-terminator expression cassette, *ACT2pt*^40^, and cloned into the binary vector pBIN19 using the *Kpn*I/*Sac*I restriction sites. Plasmids were introduced into the *Agrobacterium tumefaciens* strain C58 using the heat shock method.

*Arabidopsis* plants were transformed by vacuum infiltration as described previously^41^. Transgenic plants were selected on kanamycin and the resulting T_0_ seedlings of *Arabidopsis* expressing OsPIP1;3 were grown in soil and independent T_1_ transgenic lines were obtained. Homozygous T_2_ lines were generated by growing the T_1_ transgenic lines showing 3:1 (resistant: sensitive) Mendelian segregation ratio. Transcript analysis was performed on the T_2_ transgenic *Arabidopsis* lines by a semi-quantitative RT-PCR as described above. For studying the role of OsPIP2;6 in response to B treatment, we used the three T_2_ homozygous *Arabidopsis* lines overexpressing OsPIP2;6 (27, 33, and 40) that were generated in our previous study^32^. For B phenotypic analysis, seeds of the wild type and transgenic overexpression lines were germinated and grown on 1/2x MS agar plates containing 0 or 2.5 mM boric acid for three weeks. Shoot biomass and root lengths were determined. For aquaporin inhibitors treatment, 100 μM NaN_3_ (sodium azide) and 50 μM AgNO_3_ (silver nitrate) were used with or without boric acid.

### Boron Transport Assays in *Arabidopsis*

Wild type and transgenic *Arabidopsis* seeds were grown on a nylon mesh placed on 1/2x MS plates for two weeks with 16/8 hrs light/dark cycles at 22 °C. The nylon mesh along with seedlings was then transferred, placed on a support into the magenta boxes containing1/2x MS liquid media and plants were grown for one-week for acclimatization. For long-term B accumulation assay, the 1/2x MS liquid medium was replaced with new medium containing 2.5 mM boric acid and plants were grown for another four days. Shoot and root tissues were harvested, washed with deionized water, and dried at 70 °C for 48 hrs. Dried tissues were digested in concentrated nitric acid and hydrogen peroxide and then samples were analyzed by ICP-MS. For B influx assay, 5 mM ^10^B-enriched boric acid (99%, Cambridge Isotope Laboratories, Andover, MA, USA) was used. Boric acid concentration was selected based on the optimization of Arabidopsis plant growths on wide range of concentrations. The shoot and root tissues were harvested separately after 1, 2, and 3 hrs of ^10^B treatment. ^10^B in roots and shoots was measured by ICP-MS. For B efflux assay, plants were treated with 5 mM ^10^B-enriched boric acid for 3 hrs and washed quickly with deionized water and then transferred to 1/2x MS liquid medium without B. Roots and shoots were harvested after 0, 1, 2, and 3 hrs of transferring to B free media. ^10^B was analyzed in roots, shoots, and efflux media by ICP-MS

### Statistical Analysis

One-way ANOVA followed by least significant difference (LSD) multiple comparison test (p < 0.01 and p < 0.05) was used to determine all differences of statistical significance among treatments. Standard error of the mean were calculated and represented in all figures. Elemental content is expressed on a dry weight basis.

## Additional Information

**How to cite this article**: Mosa, K. A. *et al.* Enhanced Boron Tolerance in Plants Mediated by Bidirectional Transport Through Plasma Membrane Intrinsic Proteins. *Sci. Rep.* **6**, 21640; doi: 10.1038/srep21640 (2016).

## Supplementary Material

Supplementary Information

## References

[b1] Brown P. H. *et al.* Boron in plant biology. Plant biology 4(2), 205–223 (2002).

[b2] Reguera M., Espí A., Bolaños L., Bonilla I. & Redondo-Nieto M. Endoreduplication before cell differentiation fails in boron-deficient legume nodules. Is boron involved in signalling during cell cycle regulation? New Phytologist 183(1), 8–12 (2009). 19453431 10.1111/j.1469-8137.2009.02869.x

[b3] Pennisi M., Bianchini G., Muti A., Kloppmann W. & Gonfiantini R. Behaviour of boron and strontium isotopes in groundwater–aquifer interactions in the Cornia Plain (Tuscany, Italy). Applied Geochemistry 21(7), 1169–1183 (2006).

[b4] Nable R. O., Bañuelos G. S. & Paull J. G. Boron toxicity. Plant and Soil 193 **(1–2),** 181–198 (1997).

[b5] Reid R. Understanding the boron transport network in plants. Plant and Soil 385 **(1–2),** 1–13 (2014).

[b6] Gomes D. *et al.* Aquaporins are multifunctional water and solute transporters highly divergent in living organisms. Biochimica et Biophysica Acta 1788(6), 1213–1228 (2009). 19327343 10.1016/j.bbamem.2009.03.009

[b7] Johanson U. *et al.* The complete set of genes encoding major intrinsic proteins in Arabidopsis provides a framework for a new nomenclature for major intrinsic proteins in plants. Plant physiology 126(4), 1358–1369 (2001). 11500536 10.1104/pp.126.4.1358 PMC117137

[b8] Maurel C., Verdoucq L., Luu D. T. & Santoni V. Plant aquaporins: membrane channels with multiple integrated functions. Annu. Rev. Plant Biol. 59, 595–624 (2008). 18444909 10.1146/annurev.arplant.59.032607.092734

[b9] Danielson J. Å. & Johanson U. Unexpected complexity of the aquaporin gene family in the moss Physcomitrella patens. BMC Plant Biology 8(1), 45 (2008). 18430224 10.1186/1471-2229-8-45 PMC2386804

[b10] Bienert G. P., Bienert M. D., Jahn T. P., Boutry M. & Chaumont F. Solanaceae XIPs are plasma membrane aquaporins that facilitate the transport of many uncharged substrates. The Plant Journal 66(2), 306–317 (2011). 21241387 10.1111/j.1365-313X.2011.04496.x

[b11] Chaumont F., Barrieu F., Jung R. & Chrispeels M. J. Plasma membrane intrinsic proteins from maize cluster in two sequence subgroups with differential aquaporin activity. Plant Physiology 122(4), 1025–1034 (2000). 10759498 10.1104/pp.122.4.1025 PMC58937

[b12] Kammerloher W., Fischer U., Piechottka G. P. & Schäffner A. R. Water channels in the plant plasma membrane cloned by immunoselection from a mammalian expression system. The Plant Journal 6(2), 187–199 (1994). 7920711 10.1046/j.1365-313x.1994.6020187.x

[b13] Weig A., Deswarte C. & Chrispeels M. J. The major intrinsic protein family of Arabidopsis has 23 members that form three distinct groups with functional aquaporins in each group. Plant Physiology 114(4), 1347–1357 (1997). 9276952 10.1104/pp.114.4.1347 PMC158427

[b14] Sakurai J., Ishikawa F., Yamaguchi T., Uemura M. & Maeshima M. Identification of 33 rice aquaporin genes and analysis of their expression and function. Plant and Cell Physiology 46(9), 1568–1577 (2005). 16033806 10.1093/pcp/pci172

[b15] Takano J. *et al.* *Arabidopsis* boron transporter for xylem loading. Nature 420(6913), 337–340 (2002) 12447444 10.1038/nature01139

[b16] Miwa K., Takano J. & Fujiwara T. Improvement of seed yields under boron-limiting conditions through overexpression of BOR1, a boron transporter for xylem loading, in Arabidopsis thaliana. The Plant Journal 46(6), 1084–1091 (2006). 16805739 10.1111/j.1365-313X.2006.02763.x

[b17] Sutton T. *et al.* Boron-toxicity tolerance in barley arising from efflux transporter amplification. Science 318(5855), 1446–1449 (2007). 18048688 10.1126/science.1146853

[b18] Reid R. Identification of boron transporter genes likely to be responsible for tolerance to boron toxicity in wheat and barley. Plant and cell physiology 48(12), 1673–1678 (2007). 18003669 10.1093/pcp/pcm159

[b19] Miwa K. *et al.* Plants tolerant of high boron levels. Science 318(5855), 1417–1417 (2007). 18048682 10.1126/science.1146634

[b20] Miwa K. *et al.* Roles of BOR2, a boron exporter, in cross linking of rhamnogalacturonan II and root elongation under boron limitation in Arabidopsis. Plant physiology 163(4), 1699–1709 (2013). 24114060 10.1104/pp.113.225995 PMC3850200

[b21] Tanaka N. *et al.* Roles of pollen-specific boron efflux transporter, OsBOR4, in the rice fertilization process. Plant and Cell Physiology pct**136**. (2013). 10.1093/pcp/pct136 24068795

[b22] Takano J. *et al.* The Arabidopsis major intrinsic protein NIP5; 1 is essential for efficient boron uptake and plant development under boron limitation. The Plant Cell 18(6), 1498–1509 (2006). 16679457 10.1105/tpc.106.041640 PMC1475503

[b23] Tanaka M., Wallace I. S., Takano J., Roberts D. M. & Fujiwara T. NIP6; 1 is a boric acid channel for preferential transport of boron to growing shoot tissues in Arabidopsis. The Plant Cell 20(10), 2860–2875 (2008). 18952773 10.1105/tpc.108.058628 PMC2590723

[b24] Schnurbusch T. *et al.* Boron toxicity tolerance in barley through reduced expression of the multifunctional aquaporin HvNIP2; 1. Plant Physiology 153(4), 1706–1715 (2010). 20581256 10.1104/pp.110.158832 PMC2923888

[b25] Bogacki P., Peck D. M., Nair R. M., Howie J. & Oldach K. H. Genetic analysis of tolerance to Boron toxicity in the legume *Medicago truncatula*. BMC plant biology 13(1), 54 (2013). 23531152 10.1186/1471-2229-13-54 PMC3636127

[b26] Pang Y. *et al.* Overexpression of the tonoplast aquaporin AtTIP5; 1 conferred tolerance to boron toxicity in Arabidopsis. Journal of Genetics and Genomics 37(6), 389–397 (2010). 20621021 10.1016/S1673-8527(09)60057-6

[b27] Dordas C. & Brown P. H. Evidence for channel mediated transport of boric acid in squash (Cucurbita pepo). Plant and Soil 235(1), 95–103 (2001).

[b28] Fitzpatrick K. L. & Reid R. J. The involvement of aquaglyceroporins in transport of boron in barley roots. Plant, cell & environment 32(10), 1357–1365 (2009). 10.1111/j.1365-3040.2009.02003.x 19552667

[b29] Kumar K. *et al.* Two rice plasma membrane intrinsic proteins, OsPIP2; 4 and OsPIP2; 7, are involved in transport and providing tolerance to boron toxicity. Planta 239(1), 187–198 (2014). 24142111 10.1007/s00425-013-1969-y

[b30] Liu Z. *et al.* Arsenite transport by mammalian aquaglyceroporins AQP7 and AQP9. Proc natl acad Sci 99(9), 6053–6058 (2002). 11972053 10.1073/pnas.092131899 PMC122900

[b31] Bienert G. P. *et al.* A subgroup of plant aquaporins facilitate the bi-directional diffusion of As (OH) 3 and Sb (OH) 3 across membranes. BMC Biology 6(1), 26 (2008). 18544156 10.1186/1741-7007-6-26 PMC2442057

[b32] Mosa K. A. *et al.* Members of rice plasma membrane intrinsic proteins subfamily are involved in arsenite permeability and tolerance in plants. Transgenic research 21(6), 1265–1277 (2012). 22350764 10.1007/s11248-012-9600-8

[b33] Ma J. F. *et al.* Transporters of arsenite in rice and their role in arsenic accumulation in rice grain. Proc natl acad Sci 105(29), 9931–9935 (2008). 18626020 10.1073/pnas.0802361105 PMC2481375

[b34] Isayenkov S. V. & Maathuis F. J. The Arabidopsis thaliana aquaglyceroporin AtNIP7; 1 is a pathway for arsenite uptake. FEBS Letters 582(11), 1625–1628 (2008). 10.1016/j.febslet.2008.04.022 18435919

[b35] Kamiya T. *et al.* NIP1; 1, an aquaporin homolog, determines the arsenite sensitivity of Arabidopsis thaliana. Journal of Biological Chemistry 284(4), 2114–2120 (2009). 19029297 10.1074/jbc.M806881200

[b36] Niemietz C. M. & Tyerman S. D. New potent inhibitors of aquaporins: silver and gold compounds inhibit aquaporins of plant and human origin. FEBS Letters 531(3), 443–447 (2002). 12435590 10.1016/s0014-5793(02)03581-0

[b37] Postaire O. *et al.* A PIP1 aquaporin contributes to hydrostatic pressure-induced water transport in both the root and rosette of Arabidopsis. Plant Physiology 152(3), 1418–1430 (2010). 20034965 10.1104/pp.109.145326 PMC2832249

[b38] Mathews S., Rathinasabapathi B. & Ma L. Q. Uptake and translocation of arsenite by *Pteris vittata* L.: effects of glycerol, antimonite and silver. Environmental Pollution 159(12), 3490–3495 (2011). 21893373 10.1016/j.envpol.2011.08.027

[b39] Livak K. J. & Schmittgen T. D. Analysis of relative gene expression data using real-time quantitative PCR and the 2^−ΔΔCT^ method. methods 25(4), 402–408 (2001). 11846609 10.1006/meth.2001.1262

[b40] Dhankher O. P. *et al.* Engineering tolerance and hyperaccumulation of arsenic in plants by combining arsenate reductase and γ-glutamylcysteine synthetase expression. Nat Biotech. 20(11), 1140–1145 (2002). 10.1038/nbt747 12368812

[b41] Bechtold N. & Pelletier G. In planta *Agrobacterium*-mediated transformation of adult *Arabidopsis thaliana* plants by vacuum infiltration. In Arabidopsis protocols (pp. 259–266) Humana Press (1998). 10.1385/0-89603-391-0:259 9664431

