# Supplementary material for: Enhanced Boron Tolerance in Plants Mediated by Bidirectional Transport Through Plasma Membrane Intrinsic Proteins
Source: Sci Rep. 2016 Feb 23;6:21640. doi: 10.1038/srep21640 (PMC4763227; doi:10.1038/srep21640)
Supplement: Supplementary Information [file srep21640-s1.pdf]

Enhanced Boron Tolerance in Plants Mediated by Bidirectional Transport Through Plasma Membrane Intrinsic Proteins

Kareem A. Mosa[1,2,3] , Kundan Kumar[1,4], Sudesh Chhikara[1], Craig Musante[5], Jason C. White[5] and Om Parkash Dhankher[1*]

[1]Stockbridge School of Agriculture, University of Massachusetts, Amherst, MA 01003, USA.

[2]Department of Biotechnology, Faculty of Agriculture, Al-Azhar University, Cairo, Egypt.

[3]Department of Applied Biology, College of Science, Sharjah University, P.O. Box 27272, Sharjah, United Arab Emirates

[4]Department of Biological Sciences, Birla Institute of Technology & Science, K. K. Birla Goa Campus, Goa 403726, India.

[5]Department of Analytical Chemistry, The Connecticut Agricultural Experiment Station, New Haven, CT 06504-1106, USA

[*]**Corresponding author:** Om Parkash Dhankher (Email: parkash@umass.edu)

Supplementary Figures

Supplementary Figure S1

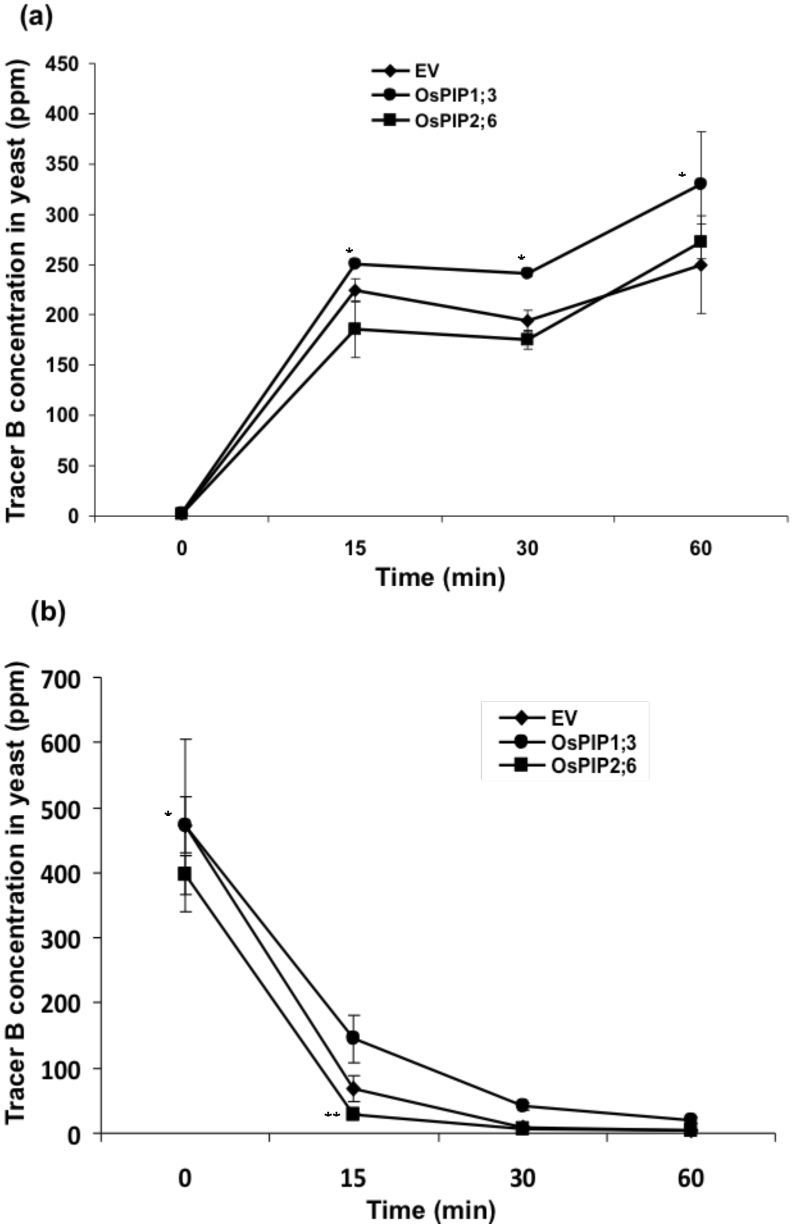

Fig. S1 Short-term assay for influx and efflux activity of OsPIP1;3 and OsPIP2;6 in *S. cerevisiae* HD9 strain (*Δfsp1Δacr3Δycf1*). (a) Concentration of tracer B (^{10}B) in the HD9 mutant yeast cells harboring the empty pYES3 plasmid (EV) or plasmids expressing t-OsPIP1;3 and t-OsPIP2;6 for short duration exposure to ^{10}B for 0, 15, 30 and 60 minutes (influx). **(b)** Concentration of ^{10}B in the HD9 mutant yeast cells harboring the empty pYES3 plasmid (EV) or plasmids expressing OsPIP1;3 and OsPIP2;6 after re-suspension in B free media for 0, 15, 30 and 60 minutes (efflux). Data are means ± S.D (n=3), (*) P<0.05.

Supplementary Figure S2

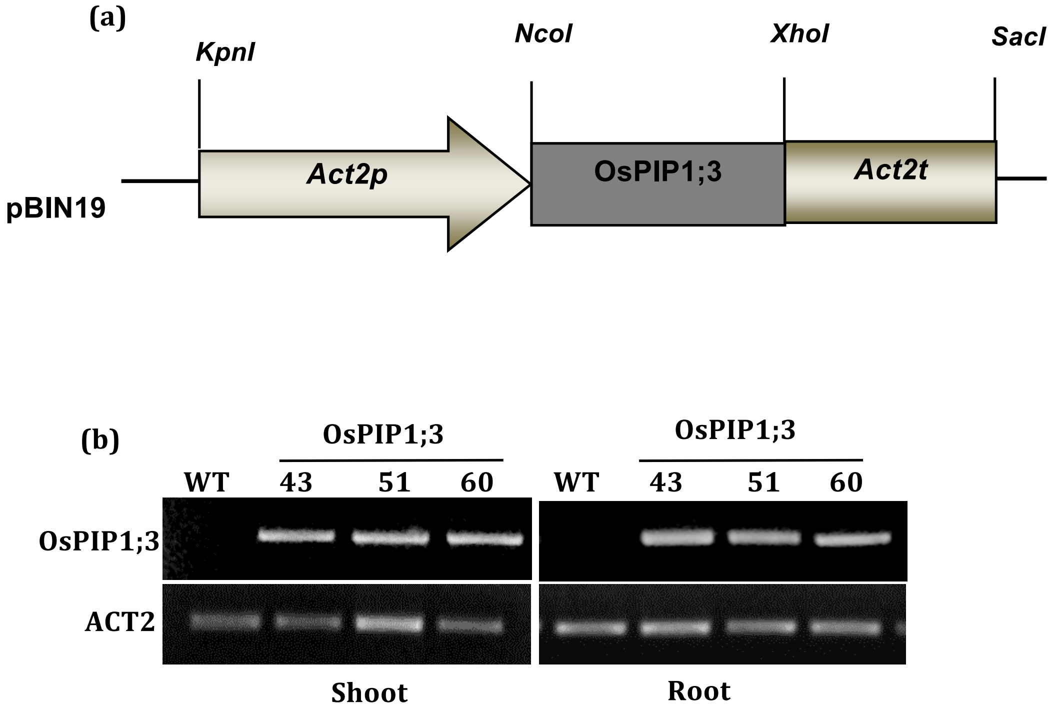

Fig. S2 Overexpression of OsPIP1;3 in *Arabidopsis*, map of overexpression construct, and RT-PCR analysis in transgenic lines. (a) Diagrams of OsPIP1;3 construct cloned under *actin2* promoter-terminator cassette (*ACT2pt*) in the binary vector pBIN19 for plant transformation. (**b)** Transcript level of OsPIP1;3 in the transgenic *Arabidopsis* lines 43, 51 and 60 in comparison with wild type control (WT). Transcriptional levels were determined using a semi-quantitative RT-PCR. *Actin2* gene (ACT2; lower panels) was used as a control.

Supplementary Figure S3

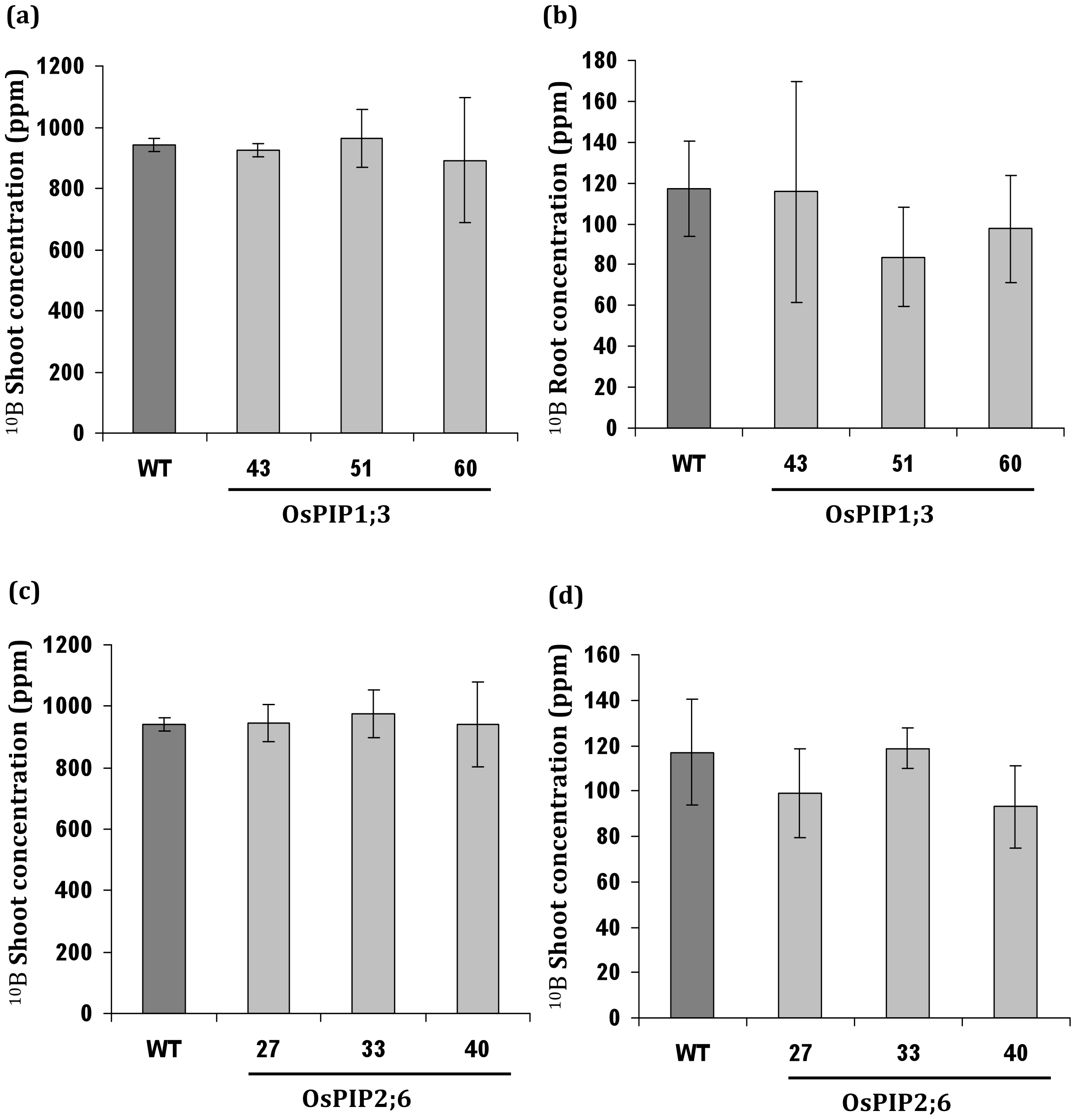

Fig. S3 Analysis of ^{10}B accumulation in the transgenic *Arabidopsis* lines expressing OsPIP1;3 and OsPIP2;6. ^{10}B concentration in **(a)** shoot and **(b)** root in the transgenic *Arabidopsis* lines 43, 51, and 60 overexpressing OsPIP1;3 in comparison with wild type (WT). ^{10}B concentration in **(c)** shoot and **(d)** in the transgenic *Arabidopsis* lines 27, 33, and 40 overexpressing OsPIP2;6 in comparison with wild type (WT). The average and standard error (SE) values are shown for three replicates of 25 plants each for WT and transgenic *Arabidopsis* lines**.**

Supplementary Figure S4

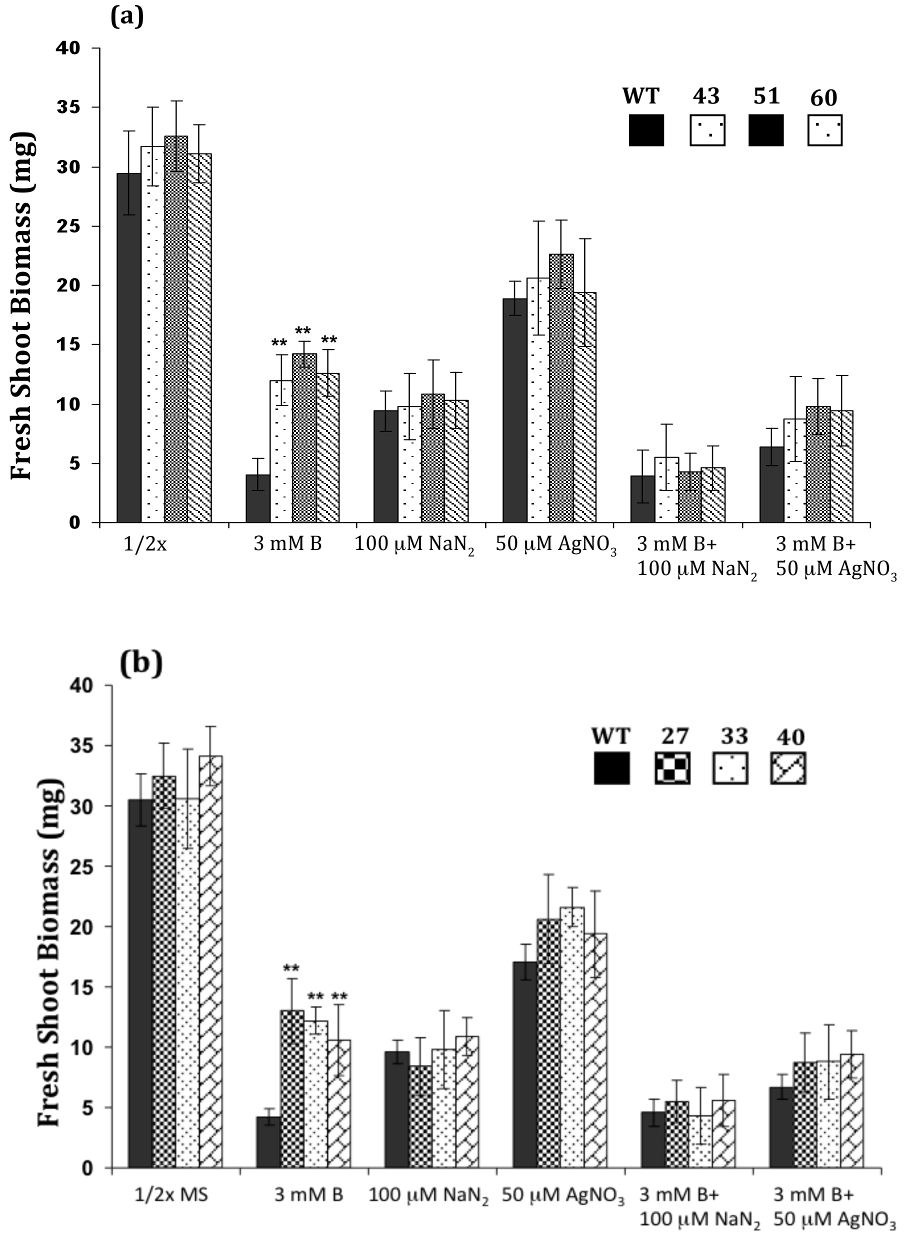

Fig. S4 Fresh shoot weight of *Arabidopsis* expressing OsPIP1;3 and OsPIP2;6 on boron containing media and aquporin transporter inhibitors. **(a)** Fresh shoot weight of the transgenic lines 43, 51 and 60 expressing OsPIP1;3 as compared with wild type (WT). **(b)** Fresh shoot weight of the transgenic lines 27, 33 and 40 expressing OsPIP2;6 as compared with wild type (WT). Seedlings were grown for 3 weeks on 1/2x MS medium supplemented with 0, 3 mM B, 100 µM sodium azide, 50 µM silver nitrate, 3 mM B+100 µM sodium azide, and 3 mM B+50 µM silver nitrate. The values are presented as an average of four replicates of 40 plants. The asterisks represent the significant difference in biomass accumulation and root length compared with wild type (WT) plants. (*) $P<0.05$, (**)
